# Sugar-Annulated Oxazoline Ligands: A Novel Pd(II) Complex and Its Application in Allylic Substitution

**DOI:** 10.3390/molecules21121704

**Published:** 2016-12-10

**Authors:** Jochen Kraft, Katharina Mill, Thomas Ziegler

**Affiliations:** Institute of Organic Chemistry, University of Tübingen, Auf der Morgenstelle 18, 72076 Tübingen, Germany; jochen.kraft@uni-tuebingen.de (J.K.); katharina.mill@student.uni-tuebingen.de (K.M.)

**Keywords:** asymmetric catalysis, carbohydrates, oxazolines, palladium complexes, pyridines, Tsuji-Trost

## Abstract

Two novel carbohydrate-derived pyridyl (PYOX)- and cyclopropyl (CYBOX)-substituted oxazoline ligands were prepared from d-glucosamine hydrochloride and 1,3,4,6-tetra-*O*-acetyl-2-amino-2-deoxy-β-d-glucopyranose hydrochloride in two steps, respectively. The sugar-annulated PYOX ligand formed a stable metal complex with Pd(II), which was fully characterized by NMR spectroscopy and X-ray crystallography. NMR and X-ray analysis revealed a change of the conformation in the sugar moiety upon complexation with the palladium(II) species. Both glycosylated ligands resulted in high asymmetric induction (up to 98% *ee*) upon application as chiral ligands in the Pd-catalyzed allylic alkylation of *rac*-1,3-diphenylallyl acetate with dimethyl malonate (Tsuji-Trost reaction). Both ligands provided mainly the (*R*)-enantiomer of the alkylation product.

## 1. Introduction

During the past decades, the use of carbohydrates as chiral auxiliaries, chiral reagents, organocatalysts and, most notably, as chiral ligands for metal-catalyzed stereoselective reactions has become a powerful tool for organic chemists [1,2]. This is in part due to the fact that carbohydrates are one of the most abundant chiral natural products on earth and, therefore, in many cases are easily isolable from natural sources in high enantiomeric purity and large quantities. As a matter of fact, several highly efficient privileged ligands for asymmetric syntheses have been obtained from carbohydrates [3,4,5,6,7]. However, the enantioselective construction of C-C bonds, in particular the synthesis of tertiary carbon stereocenters, remains an ongoing challenge for organic chemists. Over the last years, however, transition metal–catalyzed allylic alkylation (Tsuji-Trost reaction) has evolved into one of the most potent methods for synthesizing such tertiary stereocenters [8,9].

**Figure 1:**
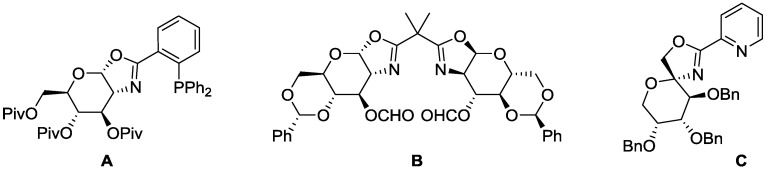
Examples of literature-known *gluco*PHOX (**A**); *gluco*BOX (**B**) and *spiro*PYOX (**C**) ligands.

In 1998, Kunz and Gläser introduced a *gluco*PHOX ligand (Figure 1A) derived from d-glucosamine, which was used in the Pd(II)-catalyzed allylic substitution of various symmetrically and non-symmetrically 1,3-disubstituted 2-propenyl acetates with dimethyl malonate [10]. The in situ generated Pd-catalyst showed a high asymmetric induction of up to 98% *ee* for the addition of dimethyl malonate to *rac*-1,3-diphenylallyl acetate.

**Figure 2:**
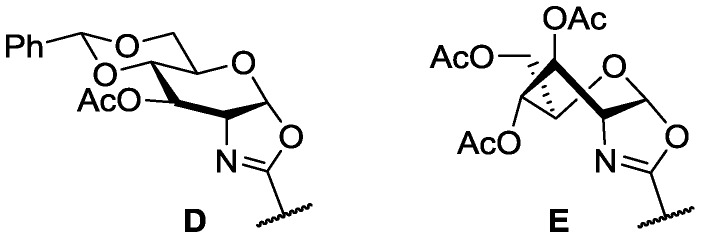
Carbohydrate conformations of sugar-annulated 1,2-oxazoline ligands in the presence (**D**) and absence (**E**) of cyclic protecting groups.

Recently, Boysen et al. described the synthesis of several d-*gluco*– and d-*allo*–configurated bisoxazoline (BOX) ligands (Figure 1B) [11,12,13]. The ligands were applied in the Cu(I)-catalyzed cyclopropanation of olefins with azoesters and in the asymmetric alkynylation of imines with up to 99% *ee*. The authors observed that a cyclic 4,6-*O*-benzylidene acetal–protecting group at the carbohydrate scaffold has a positive influence on the stereo-discrimination properties of the ligands. Boysen et al. attributed this outcome to the ^4^*H*_5_ chair-like conformation of the carbohydrate moiety (Figure 2D) which is fixed by the 4,6-*O*-benzylidene acetal [12,13]. This conformation is in contrast to similar bisoxazoline glycosides lacking a cyclic protecting group, which adopt an ^O^*S*_2_ twist-like conformation (Figure 2E).

As part of our ongoing research program towards the development of novel carbohydrate ligands for asymmetric syntheses, we previously described the preparation of a series of spiro-fused PYOX ligands (Figure 1C) and Pd(II) complexes thereof which were applied in Pd-catalyzed allylic alkylations [14,15]. X-ray crystallography of the Pd complexes revealed that the orientation of the OBn-protecting group at C-3 in these *spiro*PYOX ligands could have a major impact on the shielded side of the metal center and, thus, could result in a different stereo-discrimination in Pd-catalyzed asymmetric reactions. Indeed, we could further demonstrate that our d-fructose–based ligands mainly provided the (*R*)-enantiomer in the Pd-catalyzed allylic alkylation of 1,3-diphenylallyl acetate with dimethyl malonate whereas the d-psicose–derived ligands gave the (*S*)-enantiomer of the alkylation product instead [15].

These results on the spiro-fused carbohydrate-oxazoline ligands encouraged us to take a closer look into sugar-annulated oxazoline ligands and their metal complexes. Herein we report a straightforward synthesis of a novel d-glucosamine–derived PYOX ligand and its Pd(II) complex. Furthermore, we report on a new sugar-annulated cyclopropane-based CYBOX ligand as well as preliminary findings in the application of these ligands in the Tsuji-Trost reaction.

## 2. Results and Discussion

### 2.1. Synthesis of Oxazoline Ligands

**Scheme 1:**
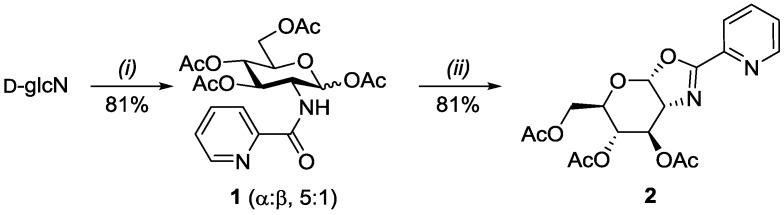
Synthesis of sugar-annulated PYOX ligand **2**. Reagents and conditions: *(**i**)* (a) HBTU/HOBt, picolinic acid, DIPEA, DMF, 0 °C-rt, 14 h; (b) Ac_2_O, pyridine, 0 °C-rt, 12 h, 81% (α:β, 5:1; determined by ^1^H-NMR); *(**ii**)* (a) HBr in AcOH, CH_2_Cl_2_, 0 °C-rt, 2 h; (b) Bu_4_NBr, NaHCO_3_, MeCN, rt, 12 h, 81%.

Our synthesis route to the PYOX ligand (Scheme 1) proceeded via amide coupling of d-glucosamine hydrochloride with picolinic acid to afford the corresponding picolinamide **1**. Subsequent cyclization of **1** under modified Lemieux conditions [11,16] gave 1,2-oxazoline **2** in 81% yield. The regioselective condensation of d-glucosamine with picolinic acid was best accomplished with the HBTU/HOBt reagent, followed by exhaustive acetylation with Ac_2_O in pyridine. Previously, glucose derivative **1** was prepared via a Pd-catalyzed aminocarbonylation of 2-pyridyl tosylate in 48% yield [17].

Although compound **1** was obtained as a 5:1 mixture of its anomers, favoring the α-anomer, the two anomers were not separated for the next synthetic step since treatment with HBr in the next step gave solely the corresponding α-glycosyl bromide intermediate from both anomers. Subsequent cyclization of the intermediate with Bu_4_NBr and NaHCO_3_ gave the annulated oxazoline ligand **2** in 81% yield.

**Scheme 2:**
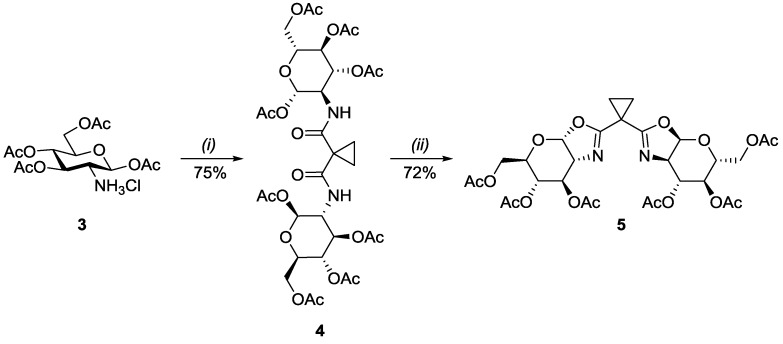
Synthesis of sugar-annulated CYBOX ligand **5**. Reagents and conditions: *(**i**)* HBTU/HOBt, cyclopropane-1,1-dicarboxylic acid, DIPEA, DMF 0 °C-rt, 15 h, 75%; *(**ii**)* (a) HBr in AcOH, CH_2_Cl_2_, 0 °C-rt, 2 h; (b) Bu_4_NBr, NaHCO_3_, MeCN rt, 17 h, 72%.

For the synthesis of the cyclopropane-based CYBOX ligand **5** (Scheme 2) we started from readily available 1,3,4,6-tetra-*O*-acetyl-2-amino-2-deoxy-β-d-glucopyranose hydrochloride **3** [18] which was condensed with cyclopropane-1,1-dicarboxylic acid under mediation of HBTU/HOBt to afford the corresponding bis-amide **4**. Cyclization to **5** was again achieved by sequential substitution of the anomeric acetate in **4** with HBr in AcOH, followed by treatment of the intermediate glycosyl bromide with Bu_4_NBr and NaHCO_3_ (Scheme 2). Yields for both steps were medium due to the formation of unidentified by-products which had to be removed by column chromatography.

### 2.2. Palladium(II) Complex of Ligand ***2***

**Scheme 3:**
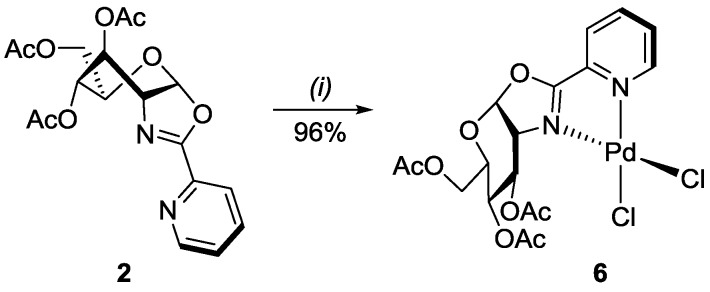
Complexation of PYOX ligand **2** with Pd(II). Reagents and conditions: *(**i**)* PdCl_2_(cod), DCE, rt, 72 h, 96%. cod = 1,5-cyclooctadiene, DCE = 1,2-dichloroethane.

The Pd(II) complex **6** was prepared by the reaction of a slight molar excess of the sugar-annulated ligand **2** with dichloro-(1,5-cyclooctadiene)palladium(II) in 1,2-dichloroethane (Scheme 3). Pd complex **6** was obtained as an orange, microcrystalline solid which was air- and moisture-stable in solution as well as in the solid state. Unfortunately, complexation of CYBOX ligand **5** with Pd(II) under identical conditions did not yield suitable crystals. Complex **6** was soluble in polar organic solvents such as MeCN, but insoluble in CH_2_Cl_2_, CHCl_3_ and non-polar organic solvents such as *n*-pentane, *n*-hexane and Et_2_O.

**Figure 3:**
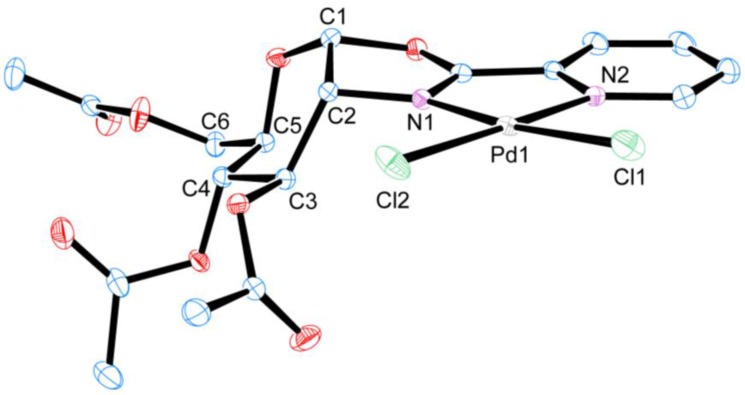
ORTEP plot of the molecular structure of the Pd complex **6**. Hydrogen atoms have been omitted for clarity; ellipsoids are given at the 50% probability level. Blue = carbon; red = oxygen; pink = nitrogen; grey = palladium; green = chlorine. Selected bond-distances (Å) and -angles (°): Pd1-N1 2.0284(14), Pd1-N2 2.0439(15), Pd-Cl1 2.2779(5), Pd-Cl2 2.2692(5); N1-Pd-N2 80.70(6).

Crystals suitable for X-ray analysis of complex **6** were obtained by slow diffusion of Et_2_O into a saturated solution of **6** in MeCN. Crystal data and structure refinements of the X-ray analysis are given in the Supplementary Material. Figure 3 illustrates the molecular structure of **6** along with selected interatomic distances and bond angles. Pd complex **6** crystallizes in the orthorhombic space group *P*2_1_2_1_2_1_ and showed the expected square-planar molecular geometry of the d^8^-configurated palladium. The bite angle of 80.7°, which is formed by oxazoline-*N* (N1) and pyridine-*N* (N2), is in accordance with previously published similar PYOX-Pd complexes [14,19,20,21].

The carbohydrate scaffold in the palladium complex **6** adopts a slightly distorted ^4^*H*_5_ conformation in the solid state. This is contrary to the conformation of similar literature-known uncomplexed 1,2-annulated sugar-oxazolines such as compound **2** which adopt a ^O^*S*_2_ conformation [22,23]. We therefore suggested a conformational change of the carbohydrate moiety upon complexation of **2** with the palladium salt. This assumption was supported by ^1^H-NMR analysis of ligand **2** and Pd complex **6** and a comparison of the relevant chemical shifts and vicinal coupling constants (Table 1). All NMR spectra were recorded in trideuteroacetonitrile in order to avoid possible solvent effects on the chemical shifts and the coupling constants.

The vicinal coupling constant *J*_3,4_ in ligand **2** changed upon complexation with PdCl_2_(cod) by 0.7 Hz, whereas *J*_4,5_ alternated by 2.0 Hz, respectively. This is in accordance with the present change of the vicinal-proton torsion angles from the modified skew (^O^*S*_2_) to the distorted half-chair (^4^*H*_5_) conformation.

### 2.3. Application of Ligands in Asymmetric Allylic Substitution

**Scheme 4:**
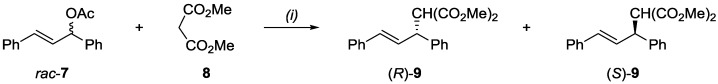
Palladium-catalyzed allylic alkylation of *rac*-1,3-diphenylallyl acetate (*rac*-**7**). Reagents and conditions: *(**i**)* Chiral ligand (**2** or **5**), [PdCl(C_3_H_5_)]_2_, KOAc/BSA, rt, 24 h. For yields and *ee*’s see Table 2. BSA = *N*,*O*-Bis(trimethylsilyl)acetamide.

Next, PYOX ligand **2** and CYBOX ligand **5** were applied as pre-catalysts in an asymmetric Tsuji-Trost reaction [8,9]. As a model system, we chose the Pd-catalyzed allylic alkylation of *rac*-1,3-diphenylallyl acetate *rac*-**7** with dimethyl malonate **8** (Scheme 4). This specific Tsuji-Trost reaction was often used as a benchmark for new carbohydrate-based ligands (Table 2, entry 1) and was investigated in great detail [10,24,25,26].

The alkylated product **9** was isolated after chromatographic purification and its enantiomeric excess was determined by chiral HPLC analysis. The absolute configuration of **9** was assigned by comparison of the optical rotation values with the literature data [27] which were based on the unambiguous configuration determination of the camphor-10-sulfonate of (*R*)-**9**, i.e., (*R*,*E*)-3,5-diphenylpent-4-enyl camphor-10-sulfonate [28]. Therefore, a positive optical rotation belongs to the (*R*)-enantiomer, whereas a negative optical rotation value refers to the (*S*)-enantiomer of **9**. Both ligands tested here were active pre-catalysts in the allylic alkylation and resulted in an excess of the (*R*)-enantiomer of **9**, as can be seen in Table 2.

The Tsuji-Trost reaction was carried out in the presence of 5 mol % [PdCl(C_3_H_5_)]_2_ and 11 mol % of the chiral ligand. The *C*_1_-symmetrical PYOX ligand **2** showed preparative yields for (*R*)-**9** of up to 92% with an enantiomeric excess of 47% (Table 2, entry 2). Running the reaction in THF or acetonitrile, respectively, yielded (*R*)-**9** in a somewhat lower yield (53%–71%) but increased the stereoselectivity of the reaction (56% *ee*, Table 2, entries 3 and 4). Changing the solvent to toluene inhibited the reaction nearly completely. In fact, only traces of the alkylated product could be obtained in toluene (Table 2, entry 5). Lowering the temperature of the reaction slightly increased the selectivity (66% *ee*) but yielded (*R*)-**9** at only 9% (Table 2, entry 6). The use of preformed Pd(II) complex **6** and in situ dehalogenation with AgSbF_6_ showed no improvement in conversion (38%) and enantioselectivity (47% *ee*, Table 2, entry 7).

To our delight, *C*_2_-symmetrical CYBOX ligand **5** showed a high asymmetric induction of up to 98% *ee* when used as a pre-catalyst in the allylic alkylation of *rac*-**7** (Table 2, entry 8). Unfortunately, by switching the solvent to THF or toluene, the reaction was found to be completely blocked (Table 2, entries 9 and 10). Running the reaction in acetonitrile slightly decreased the conversion (35%), although the enantioselectivity remained excellent (97% *ee*, Table 2, entry 11).

**Scheme 5:**
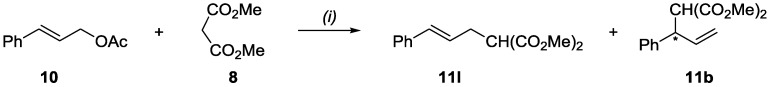
Palladium-catalyzed allylic alkylation of cinnamyl acetate (**10**). Reagents and conditions: *(**i**)* CYBOX ligand **5**, [PdCl(C_3_H_5_)]_2_, KOAc/BSA, MeCN, rt, 24 h, 100% conv., **11l**:**11b** ≥ 99:1. BSA = *N*,*O*-Bis(trimethylsilyl)acetamide.

Encouraged by these results we decided to investigate the regioselectivity and enantioselectivity of the allylic substitution of non-symmetrical substituted cinnamyl acetate **10** with dimethyl malonate **8** (Scheme 5). This catalytic system was previously studied in great detail by Pfaltz and coworkers [29]. Generally, monosubstituted allylic substrates such as **10** react predominantly at the unsubstituted allyl terminus to give the achiral, linear product **11l** rather than the chiral, branched regioisomer **11b** [30].

As very low conversions (5%–7%) were found within 24 h of reaction time with 5 mol % of [PdCl(C_3_H_5_)]_2_ and 11 mol % of PYOX ligand **2**, we decided to conduct further studies with CYBOX ligand **5**. However, cyclopropane-based ligand **5** led to full conversions in 24 h and afforded nearly exclusively linear substituted product **11l** (**11l**:**11b** ≥ 99:1).

### 2.4. Proposed Mechanism

**Scheme 6:**
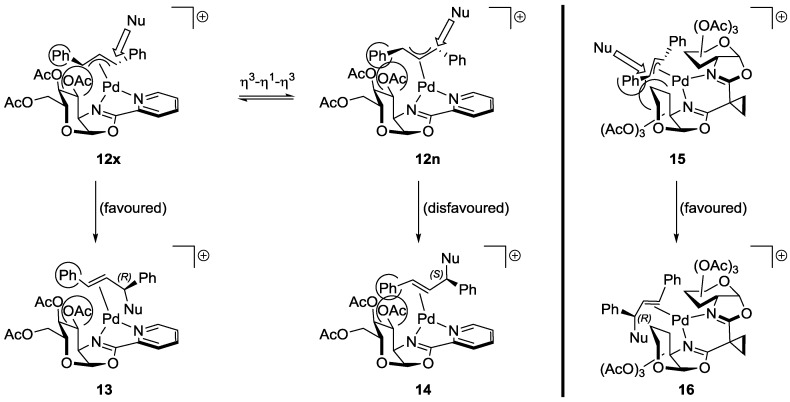
Proposed transition state and origin of enantio-discrimination in allylic substitution. Nu = CH(CO_2_Me).

The stereoselective outcome of the Tsuji-Trost reaction can be explained by a model for the proposed transition state (Scheme 6). Due to the *C*_1_-symmetry of PYOX ligand **2**, *exo* (**12x**) and *endo* (**12n**) diastereomers of the intermediate allyl-palladium complexes can be distinguished. These *exo*/*endo* diastereomers exist in a dynamic equilibrium and can isomerize via a fast η^3^–η^1^–η^3^ mechanism. The *exo*/*endo* isomerization is approximately 10 to 100 times faster than the nucleophilic addition to the allyl-palladium complex. Thus, it is reasonable to assume that the *exo*/*endo* ratios have a major impact on the observed stereoselectivites [9,26]. The attack of the nucleophile is therefore possible in four different ways: at the allyl terminus which is *cis* or *trans* to the oxazoline ring in each of the *exo* or *endo* diastereomers, respectively. In accordance with previously published reports of PYOX ligands in allylic substitutions, we assume that the attack of the nucleophile occurs at the allyl terminus which is *trans* to the oxazoline [31,32]. If the nucleophile attacks *trans* to the oxazoline ring in the *endo* complex **12n**, the formed η^2^ complex **14** must have the (*S*) configuration which, however, is contrary to our observed stereoselectivity. Hence, we suggest that the nucleophilic attack occurs predominantly to the *exo* isomer **12x**, leading to the η^2^ complex **13** which exhibits the (*R*) configuration.

In the case of the *C*_2_-symmetrical CYBOX ligand **5**, there is no distinction between *exo* and *endo* diastereomers in the allyl-palladium intermediate **15**. Therefore, there are only two possible reaction pathways. The origin of the enantio-discrimination arises here from repulsive interactions between one of the carbohydrate moieties and a phenyl group in the allyl substrate [33]. If the nucleophilic attack occurs at the allyl terminus at which the repulsion between the carbohydrate scaffold and the phenylgroup is present, the steric strain in **15** will be reduced. The so-formed η^2^ complex **16** exhibits an (*R*) configuration, which is in accordance with our observed stereoselectivity.

## 3. Materials and Methods

### 3.1. General Remarks

All solvents were dried according to standard methods, distilled and stored over molecular sieves 3 Å under an atmosphere of nitrogen prior to their use. Petroleum ether (PE) refers to the fraction boiling in the 60–90 °C range. All non-aqueous reactions were performed in oven-dried glassware under an atmosphere of N_2_ unless stated otherwise. NMR spectra were recorded on a Bruker Avance 400 spectrometer (Bruker Biospin GmbH, Rheinstetten, Germany) and were calibrated for the solvent signal (^1^H-CDCl_3_: 7.26 ppm; ^13^C-CDCl_3_: 77.16 ppm. ^1^H-CD_3_CN: 1.94 ppm; ^13^C-CD_3_CN: 1.32 ppm). NMR signals were numbered in accordance with carbohydrate nomenclature. ESI-HRMS data were measured on a Bruker Daltonics MAXIS 4G spectrometer (Bruker Daltonics GmbH, Bremen, Germany). MALDI-TOF spectra were recorded on a Bruker Autoflex II (Bruker Daltonics GmbH) using *trans*-2-[3-(4-*tert*-butylphenyl)-2-methyl-2-propylidene]malonodinitril (DCTB) as the matrix. Elemental analyses were performed on a HEKAtech Euro 3000 CHN (HEKAtech GmbH, Wegberg, Germany) analyzer. Optical rotations were measured with a Perkin-Elmer Polarimeter 341 (Perkin Elmer Inc., Waltham, MA, USA) in a 10 cm cuvette at 20 °C. Melting points were determined with a Büchi Melting Point M-560 apparatus (BÜCHI Labortechnik GmbH, Essen, Germany). Reactions were monitored by TLC on Polygram Sil G/UV silica gel plates from Machery & Nagel (Macherey & Nagel GmbH & Co. KG, Düren, Germany). Detection of spots was effected by charring with H_2_SO_4_ (5% in EtOH), staining by spraying the plates with an alkaline aqueous solution of potassium permanganate or by inspection of the TLC plates under UV light. Preparative chromatography was performed on silica gel (0.032–0.063 mm) from Machery & Nagel (Macherey & Nagel GmbH & Co. KG) with different mixtures of solvents as eluent. Racemic samples of **9** and **11** were synthesized according to the general procedure described in Section 3.4. by using 4,5-Bis(diphenylphosphino)-9,9-dimethylxanthene (Xantphos) as ligand. The enantiomeric excess of compound **9** was determined by chiral HPLC analysis on a Sykam S 1121 chromatograph (SYKAM Chromatographie Vertriebs GmbH, Fürstenfeldbruck, Germany) equipped with a Reprosil Chiral-NR column (*n*-hexane:*i*PrOH, 90:10; flow 1.6 mL/min): *t*_R_ = 6.8 min for (*R*)-**9**, *t*_R_ = 8.6 min for (*S*)-**9**. All yields given below are isolated yields determined after purification of the product either by silica gel column chromatography or crystallization and were not optimized unless noted otherwise.

### 3.2. Synthesis of Compounds

*1,3,4,6-Tetra-O-acetyl-2-deoxy-2-picolinamido-d-glucopyranose* (**1**): To an ice cold solution of d-glucosamine hydrochloride (0.50 g, 2.32 mmol) and Hünig’s base (*N,N*-diisopropylamine) (1.20 mL, 6.96 mmol) in dry DMF (15 mL) was added in the following order: HOBt (1-hydroxybenzotriazole) (0.53 g, 3.48 mmol), picolinic acid (0.29 g, 2.32 mmol) and HBTU (2-(1*H*-benzotriazol-1-yl)-1,1,3,3-tetramethyluronium hexafluorophosphate) (1.32 g, 3.48 mmol). The resulting mixture was stirred for 1 h at 0 °C followed by rt until TLC (CHCl_3_:MeOH, 5:1) showed complete consumption of starting material (14 h). The solution was evaporated in vacuo, re-dissolved in pyridine (10 mL) and treated with Ac_2_O (1.75 mL, 18.6 mmol) at 0 °C. The resulting mixture was stirred at rt until TLC (eluent EtOAc) indicated complete *O*-acetylation (12 h). The solution was poured into ice/water mixture (50 mL) and extracted twice with CH_2_Cl_2_ (20 mL). The combined organic phases were washed with 0.5 M citric acid, sat. aqueous NaHCO_3_-sol., H_2_O and brine, dried over MgSO_4_, filtered and concentrated. Chromatography of the residue (PE:EtOAc, 1:1) afforded **1** (0.85 g, 81%) as an amorphous foam (anomeric mixture; α:β, 5:1). Spectroscopic data were in accordance with literature values [17].

*2-(2-Pyridyl)-(3,4,6-tri-O-acetyl-1,2-dideoxy-α-d-glucopyranoso)-[2,1-d]-2-oxazoline* (**2**): HBr (33% in AcOH, 4.80 mL, 27.4 mmol) was added dropwise to an icecold solution of **1** (0.73 g, 1.61 mmol) in CH_2_Cl_2_ (20 mL). After complete addition the mixture was allowed to warm to rt and stirring at this temperature was continued for 2 h until all starting material was consumed (TLC; PE:EtOAc, 1:1). The solution was hydrolyzed by addition of ice/water and the phases were separated. The organic layer was washed with sat. aqueous NaHCO_3_-sol., H_2_O and brine, dried over Na_2_SO_4_, filtered and concentrated. The residue was re-dissolved in MeCN (20 mL) and Bu_4_NBr (0.57 g, 1.77 mmol) and NaHCO_3_ (0.27 g, 3.23 mmol) were added. After stirring for additional 12 h at rt the suspension was evaporated in vacuo and the yellowish residue re-dissolved in EtOAc (15 mL), washed with H_2_O and brine, dried over Na_2_SO_4_, filtered and concentrated. Chromatography of the residue (PE:EtOAc, 1:3 +2.5% Et_3_N) afforded **2** (0.51 g, 81%) as a colorless foam. ${\left[\alpha \right]}_{D}^{20}$ +50.3 (c 1.00, CHCl_3_). R*_f_* = 0.43 (PE-EtOAc, 1:3 +2.5% Et_3_N). ^1^H-NMR (400 MHz, CDCl_3_): δ = 8.72–8.71 (m, 1H, pyridine-H), 8.04 (d, *J* = 7.9 Hz, 1H, pyridine-H), 7.82–7.80 (td, *J* = 3.9 Hz, *J* = 1.8 Hz, 1H, pyridine-H), 7.44–7.41 (m, 1H, pyridine-H), 6.23 (d, *J*_1,2_ = 7.4 Hz, 1H, H-1), 5.41 (t, *J* = 2.5 Hz, 1H, H-3), 4.97–4.94 (m, 1H, H-4), 4.42–4.39 (m, 1H, H-2), 4.15–4.13 (m, 2H, H-6a, H-6b), 3.70–3.66 (m, 1H, H-5), 2.10, 2.03, 1.98 (3s, 9H, CH_3_). ^13^C-NMR (100 MHz, CDCl_3_): δ = 170.6, 169.6, 169.2 (C=O), 164.2 (OCN), 150.2, 145.4, 136.9, 126.4, 124.3 (pyridine-C), 100.4 (C-1), 70.3 (C-3), 68.4 (C-4), 67.8 (C-5), 65.4 (C-2), 63.2 (C-6), 21.0, 20.8, 20.8 (CH_3_). HRMS-ESI: *m*/*z* calcd for C_18_H_20_N_2_O_8_Na [M + Na]^+^: 415.111187; found: 415.111056. Anal. calcd for C_18_H_20_N_2_O_8_ (392.4): C, 55.10; H, 5.14; N, 7.14; found: C, 55.06; H, 5.45; N, 6.67.

*N,N′-Bis(1,3,4,6-tetra-O-acetyl-2-deoxy-β-d-glucopyranos-2-yl)-cyclopropane-1,1-dicarboxamide* (**4**): To an ice cold solution of 1,3,4,6-tetra-*O*-acetyl-2-amino-2-deoxy-β-d-glucopyranose hydrochloride (**3**) [18] (1.20 g, 3.12 mmol) and Hünig’s base (1.63 mL, 9.38 mmol) in dry DMF (15 mL) was added in the following order: HOBt (0.72 g, 4.69 mmol), cyclopropane-1,1-dicarboxylic acid (0.20 g, 1.56 mmol) and HBTU (1.78 g, 4.69 mmol). The resulting mixture was stirred for 1 h at 0 °C followed by rt for 14 h. The mixture was evaporated to dryness and the residue re-dissolved in EtOAc (15 mL). The organic solution was washed with 0.5 m citric acid, sat. aqueous NaHCO_3_-sol., H_2_O and brine, dried over MgSO_4_, filtered and concentrated. Chromatography of the residue (PE:EtOAc, 1:3 +1% Et_3_N) afforded **4** (0.93 g, 75%) as a colorless foam. ${\left[\alpha \right]}_{D}^{20}$ +38.8 (c 1.00, CHCl_3_). R*_f_* = 0.29 (PE:EtOAc, 1:3 +1% Et_3_N). ^1^H-NMR (400 MHz, CDCl_3_): δ = 6.59 (d, *J* = 9.6 Hz, 2H, NH), 6.01 (d, *J*_1,2_ = 8.6 Hz, 2H, H-1), 5.39 (t, *J* = 9.8 Hz, 2H, H-3), 5.20 (t, *J* = 9.9 Hz, 2H, H-4), 4.37 (q, *J*_2,3_ = 9.5 Hz, 2H, H-2), 4.27 (dd, *J*_6a,6b_ = 12.6 Hz, *J*_6a,5_ = 4.8 Hz, 2H, H-6a), 4.14 (dd, *J*_6b,6a_ = 12.5 Hz, *J*_6b,5_ = 2.4 Hz, 2H, H-6b), 3.86 (ddd, *J*_5,6a_ = 4.7 Hz, *J*_5,6b_ = 2.2 Hz, *J*_5,4_ = 10.1 Hz, 2H, H-5), 2.10, 2.10, 2.08, 2.08 (4s, 24H, CH_3_), 1.54–1.49 (m, 2H, cyclopropane-CH_2_), 1.10–1.05 (m, 2H, cyclopropane-CH_2_). ^13^C-NMR (100 MHz, CDCl_3_): δ = 173.0, 170.9, 169.8, 169.3 (C=O), 168.9 (CONH), 92.5 (C-1), 74.0 (C-3), 72.9 (C-5), 67.6 (C-4), 61.8 (C-6), 53.8 (C-2), 30.3 (quar. cyclopropane-C), 21.0, 20.9, 20.8 (CH_3_), 17.5 (cyclopropane-CH_2_). HRMS-ESI: *m*/*z* calcd for C_33_H_44_N_2_O_20_Na [M + Na]^+^: 811.23796; found: 811.23806. Anal. calcd for C_33_H_44_N_2_O_20_ (788.7): C, 50.25; H, 5.62; N, 3.55; found: C, 50.28; H, 5.72; N, 3.37.

*2,2′-Cyclopropylidenbis[(3,4,6-tri-O-acetyl-1,2-dideoxy-α-d-glucopyranoso)[2,1-d]-2-oxazoline]* (**5**): HBr (33% in AcOH, 6.80 mL, 37.50 mmol) was added dropwise to an icecold solution of **4** (0.87 g, 1.10 mmol) in CH_2_Cl_2_ (15 mL). After complete addition the mixture was allowed to warm to rt and stirring at this temperature was continued for 2 h (TLC; PE:EtOAc, 1:1). The solution was hydrolyzed with ice/water mixture and the phases were separated. The organic layer was washed with sat. aqueous NaHCO_3_-sol., H_2_O and brine, dried over Na_2_SO_4_, filtered and concentrated. The residue was re-dissolved in MeCN (15 mL) and Bu_4_NBr (0.39 g, 1.21 mmol) and NaHCO_3_ (0.19 g, 2.21 mmol) were added. After stirring for additional 17 h at rt the suspension was evaporated *in vacuo* and the yellowish residue was re-dissolved in EtOAc (20 mL). The organic solution was then washed with H_2_O and brine, dried over Na_2_SO_4_, filtered and concentrated. Chromatography of the residue (PE:EtOAc, 1:3 +2.5% Et_3_N) afforded **5** (0.53 g, 72%) as a colorless foam. ${\left[\alpha \right]}_{D}^{20}$ +47.4 (c 1.00, CHCl_3_). R*_f_* = 0.38 (PE:EtOAc, 1:3 +2.5% Et_3_N). ^1^H-NMR (400 MHz, CDCl_3_): δ = 6.01 (d, *J*_1,2_ = 7.3 Hz, 2H, H-1), 5.27–5.24 (m, 2H, H-3), 4.92 (dd, *J* = 0.8 Hz, *J* = 9.4 Hz, 2H, H-4), 4.21–4.12 (m, 6H, H-2, H-6a, H-6b), 3.92–3.87 (m, 2H, H-5), 2.09, 2.08, 2.07 (3s, 18H, CH3), 1.69–1.65 (m, 2H, cyclopropane-CH_2_), 1.42–1.37 (m, 2H, cyclopropane-CH_2_). ^13^C-NMR (100 MHz, CDCl_3_): δ = 170.8 (OCN), 169.7, 169.3, 167.1 (C=O), 100.1 (C-1), 70.4 (C-3), 68.5 (C-4), 67.8 (C-5), 64.9 (C-2), 63.2 (C-6), 21.0, 20.8 (CH_3_), 18.4 (quar. cyclopropane-C), 15.7 (cyclopropane-CH_2_). HRMS-ESI: *m*/*z* calcd for C_29_H_36_N_2_O_16_Na [M + Na]^+^: 691.19570; found: 691.19644. Anal. calcd for C_29_H_36_N_2_O_16_ (668.6): C, 52.10; H, 5.43; N, 4.19; found: C, 52.35; H, 5.66; N, 4.10.

*Dichloro[2-(2-Pyridyl)-(3,4,6-tri-O-acetyl-1,2-dideoxy-α-d-glucopyranoso)-[2,1-d]-2-oxazoline]palladium(II)* (**6**): PdCl_2_(cod) (dichloro-(1,5-cyclooctadiene)palladium(II)) (57 mg, 0.200 mmol, 98 mol %) was added to a solution of **2** (80 mg, 0.204 mmol) in 4 mL dry Cl(CH_2_)_2_Cl and stirred for 72 h at rt. Evaporation to dryness and re-crystallization of the residue from dry MeCN/Et_2_O afforded the palladium complex **6** (110 mg, 96%) as orange needles. M.p. > 209 °C (decomp, Et_2_O/MeCN). ${\left[\alpha \right]}_{D}^{20}$ −143.4 (c 1.00, MeCN). ^1^H-NMR (400 MHz, CD_3_CN): δ = 8.97–8.95 (m, 1H, pyridine-H), 8.24 (td, *J* = 3.9 Hz, *J* = 1.4 Hz, 1H, pyridine-H), 7.97 (dd, *J* = 7.8 Hz, *J* = 0.9 Hz, 1H, pyridine-H), 7.80–7.77 (m, 1H, pyridine-H), 6.71 (d, *J*_1,2_ = 7.6 Hz, 1H, H-1), 6.09 (t, *J* = 3.0 Hz, 1H, H-3), 4.95 (ddd, *J* = 7.1 Hz, *J* = 2.7 Hz, *J* = 1.3 Hz, 1H, H-4), 4.67 (ddd, *J* = 7.6 Hz, *J* = 3.3 Hz, *J* = 1.3 Hz, 1H, H-2), 4.26–4.17 (m, 2H, H-6a, H-6b), 3.94–3.90 (m, 1H, H-5), 2.16, 2.07, 2.02 (CH_3_). ^13^C-NMR (100 MHz, CD_3_CN): δ = 171.9, 171.3, 170.4, 169.8 (C=O, OCN), 151.7, 145.1, 142.1, 131.2, 128.0 (pyridine-C), 106.3 (C-1), 70.6 (C-5), 67.6 (C-4), 66.9 (C-3), 64.1 (C-6), 60.9 (C-2), 21.6, 21.2, 20.9 (CH_3_). MS (MALDI-TOF): *m*/*z* = 608.9 [M + K]^+^. Anal. calcd for C_18_H_20_Cl_2_N_2_O_8_Pd (569.7): C, 37.95; H, 3.54; N, 4.92; found: C, 37.66; H, 3.45; N, 4.93. Crystal Data for C_18_H_20_Cl_2_N_2_O_8_Pd (M = 569.66 g/mol): orthorombic, space group P2_1_2_1_2_1_ (no. 19), a = 8.6185(6) Å, b = 14.6441(10) Å, c = 16.5033(11) Å, α = β = γ = 90°, V = 2082.9(2) Å^3^, Z = 4, T = 100(2) K, μ(MoK_α_) = 1.197 mm^−1^, D_calc_ = 1.817 g/cm^3^, 44885 reflections measured (2.666° ≤ 2θ ≤ 30.526°), 6370 unique (R_int_ = 0.0287) which were used in all calculations. The final R_1_ was 0.0144 (I > 2σ(I)) and wR_2_ was 0.0362 (all data).

### 3.3. Crystallography

Crystals of **6** for X-ray analysis were obtained by slow diffusion of Et_2_O into a sat. solution of **6** in MeCN. X-ray data were collected on a Bruker SMART APEX II DUO (Bruker AXS Advanced X-ray Solutions GmbH, Karlsruhe, Germany) diffractometer using a graphite-monochromated Mo K_α_ radiation (λ = 0.71073 Å). Corrections for absorption effects were applied using SADABS [34]. The structure was solved by direct methods using SHELXS and SHELXL for structure solution and refinement [35,36,37,38]. Further details of the refinement and crystallographic data are given in the supplementary material.

### 3.4. Asymmetric Allylic Alkylation (Tsuji-Trost Reaction)

General Procedure for the addition of dimethyl malonate (**8**) to allyl acetates (*rac*-**7** or **10**): A solution of chiral ligand **2** or **5** (0.011 mmol, 11 mol % in respect to the allyl acetate) and [PdCl(C_3_H_5_)]_2_ (1.8 mg, 0.005 mmol, 5 mol %) in dry CH_2_Cl_2_ (1 mL) were stirred for 30 min at rt. To this catalyst solution allyl acetate (0.1 mmol, 25 mg for *rac*-**7**; 17 µL for **10**), dimethyl malonate (**8**) (34 µL, 0.3 mmol), *N*,*O*-bis(trimethylsilyl)acetamide (73 µL, 0.3 mmol) and KOAc (0.5 mg, 0.005 mmol) were added and stirring was continued for 12 h at rt. The reaction mixture was diluted with Et_2_O (3 mL) and NH_4_Cl (2 mL) and the aqueous layer was extracted with Et_2_O (3 × 5 mL). The combined organic phases were washed with water and brine, dried over Na_2_SO_4_ and concentrated. Chromatography of the residue (PE:EtOAc, 6:1) afforded the alkylated product **9** or **11**, respectively, as a colorless oil. Spectroscopic data were in accordance with literature [39,40].

## 4. Conclusions

In summary, we have synthesized the novel sugar-annulated ligands PYOX (**2**) and CYBOX (**5**) from d-glucosamine hydrochloride and readily available 1,3,4,6-tetra-*O*-acetyl-2-amino-2-deoxy-β-d-glucopyranose hydrochloride in two steps, respectively. PYOX ligand **2** formed a stable Pd(II) complex upon reaction with PdCl_2_(cod) and was fully characterized by means of NMR, CHN, MS and crystal structure analysis. NMR and X-ray studies revealed a conformational change of the carbohydrate scaffold in ligand **2** upon complexation with Pd(II). Both ligands were used as pre-catalysts in the Pd-catalyzed allylic substitution of *rac*-1,3-diphenylallyl acetate with dimethyl malonate (Tsuji-Trost reaction). CYBOX ligand **5** provided the (*R*)-enantiomer of the alkylation product with high enantiomeric excess (up to 98% *ee*). Further ligand optimizations and substrate scope evaluations of allylic substitution are currently under investigation and will be published soon.

## Figures and Tables

**Table 1:** ^1^H-NMR comparison between uncomplexed ligand **2** and Pd complex **6**.

	2 ^a O^*S*_2_	6 ^a^ ^4^*H*_5_
***Proton chemical shifts (ppm)***		
**H-1**	6.23	6.71
**H-2**	4.44	4.67
**H-3**	5.29	6.09
**H-4**	4.92	4.95
**H-5**	3.65	3.92
**H-6a**	4.13–4.06	4.26–4.17
**H-6b**		
***Vicinal coupling constants (Hz)***		
***J*_1,2_**	7.4	7.6
***J*_2,3_**	2.7	3.3
***J*_2,4_**	1.2	1.3
***J*_3,4_**	2.3	3.0
***J*_4,5_**	9.1	7.1

^a^ ^1^H-NMR spectra were recorded at 400 MHz in CD_3_CN.

**Table 2:** Pd-catalyzed allylic alkylation of *rac*-**7** using ligands **2** and **5**.

Entry	Ligand	Solvent	Yield ^b^	*ee* ^c^
1 ^a^	A	CH_2_Cl_2_	94%	98% (*R*)
2	2	CH_2_Cl_2_	92%	47% (*R*)
3	2	THF	53%	56% (*R*)
4	2	MeCN	71%	56% (*R*)
5	2	PhCH_3_	traces	n.d.
6 ^d^	2	MeCN	9%	66% (*R*)
7 ^e,f^	2	MeCN	38%	47% (*R*)
8	5	CH_2_Cl_2_	48%	98% (*R*)
9	5	THF	traces	n.d.
10	5	PhCH_3_	traces	n.d.
11	5	MeCN	35%	97% (*R*)

^a^ Taken from Reference [10]. ^b^ Isolated yield after chromatographic purification. ^c^ Enantiomeric excess was determined by chiral HPLC analysis. The absolute configuration was determined by comparison of the optical rotation with literature values [27]. ^d^ T = 0 °C. ^e^ Preformed Pd complex **6** was used. ^f^ 10 mol % AgSbF_6_. n.d. = not determined.

## Supplementary Materials

The following are available online at http://www.mdpi.com/1420-3049/21/12/1704/s1.
