# Supplementary material for: Sugar-Annulated Oxazoline Ligands: A Novel Pd(II) Complex and Its Application in Allylic Substitution
Source: Molecules. 2016 Dec 10;21(12):1704. doi: 10.3390/molecules21121704 (PMC6274480; doi:10.3390/molecules21121704)
Supplement: Supplementary file 1 [file molecules-21-01704-s001.pdf]

Supplementary Materials: Sugar-Annulated Oxazoline Ligands: A Novel Pd(II) Complex and Its Application in Allylic Substitution

Jochen Kraft, Katharina Mill and Thomas Ziegler

Copies of NMR spectra and Crystal Structure Data

Content

NMR Spectra S1–S4

Crystal Structure Data S5–S10

NMR Spectra

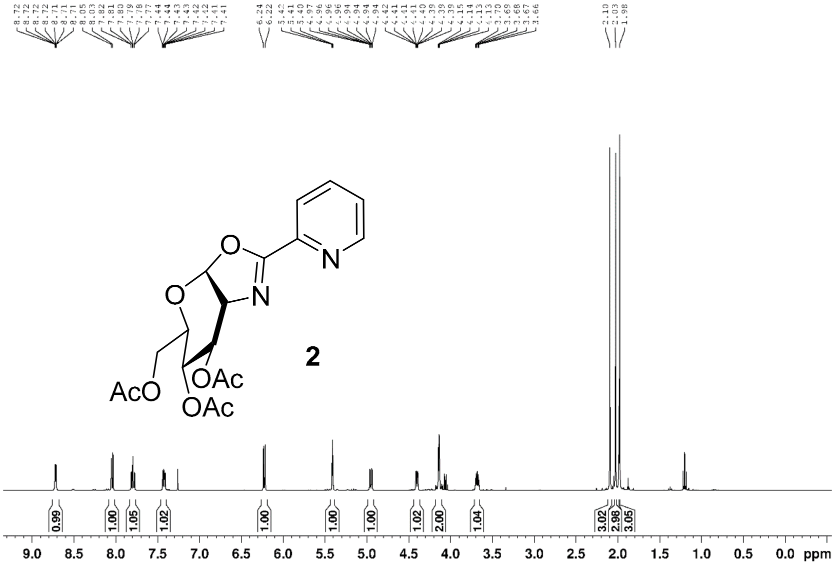

Figure S1. ^{1}H-NMR of compound **2**.

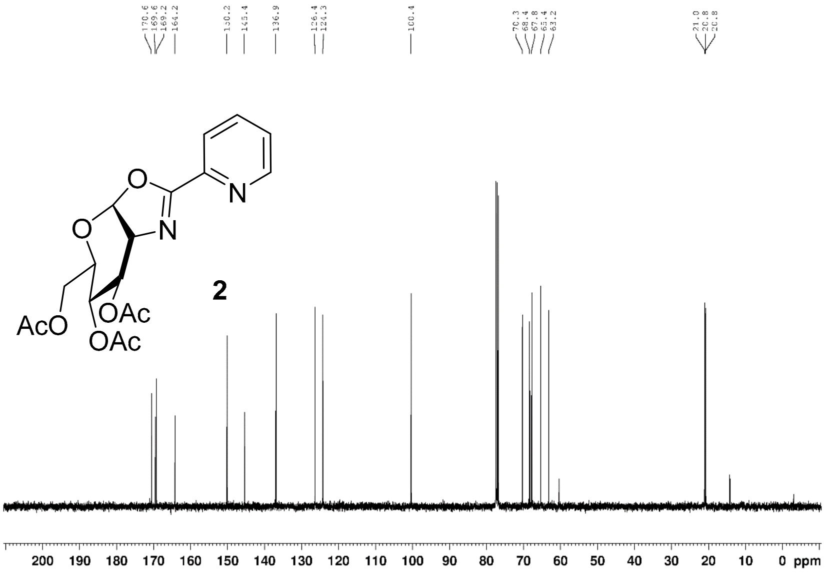

Figure S2. ^{13}C-NMR of compound **2**.

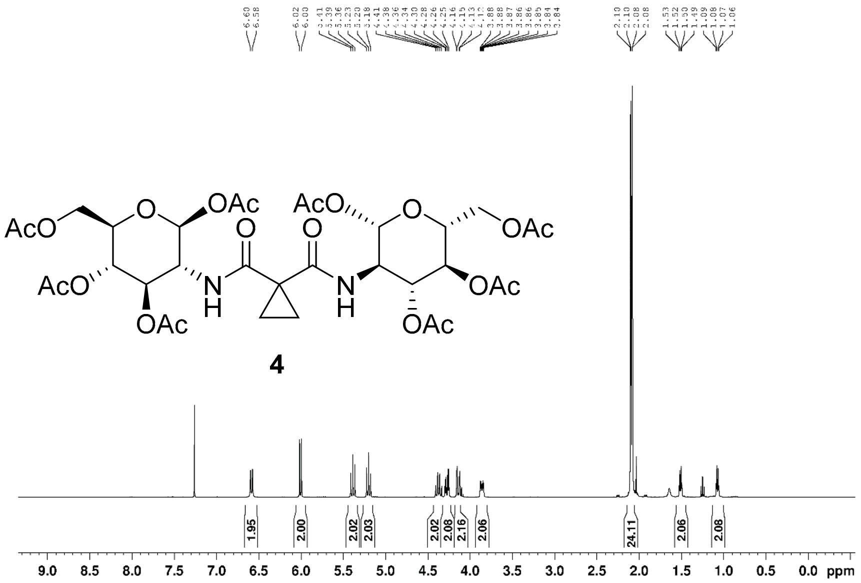

Figure S3. ^{1}H-NMR of compound **4**.

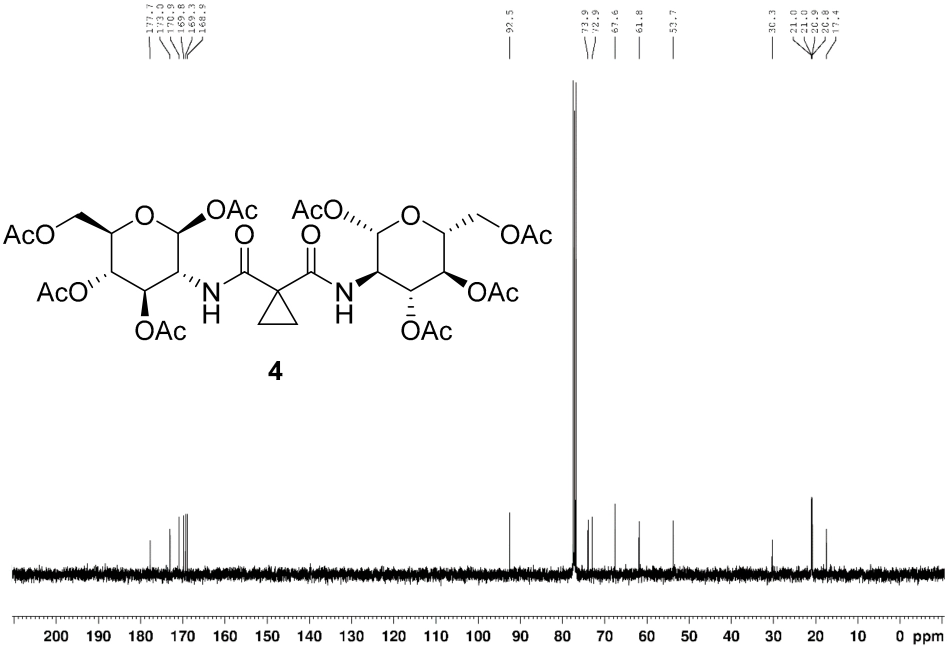

Figure S4. ^{13}C-NMR of compound **4**.

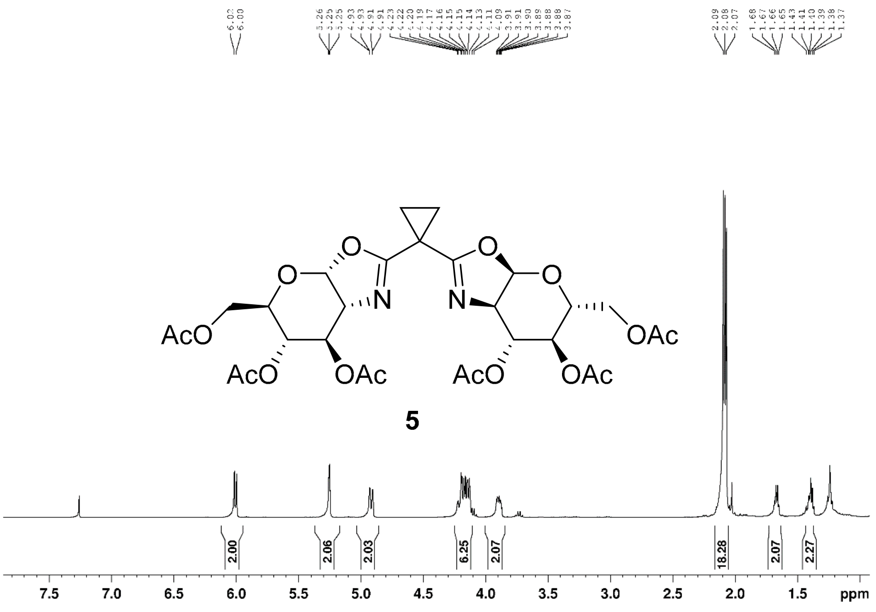

Figure S5. ^{1}H-NMR of compound **5**.

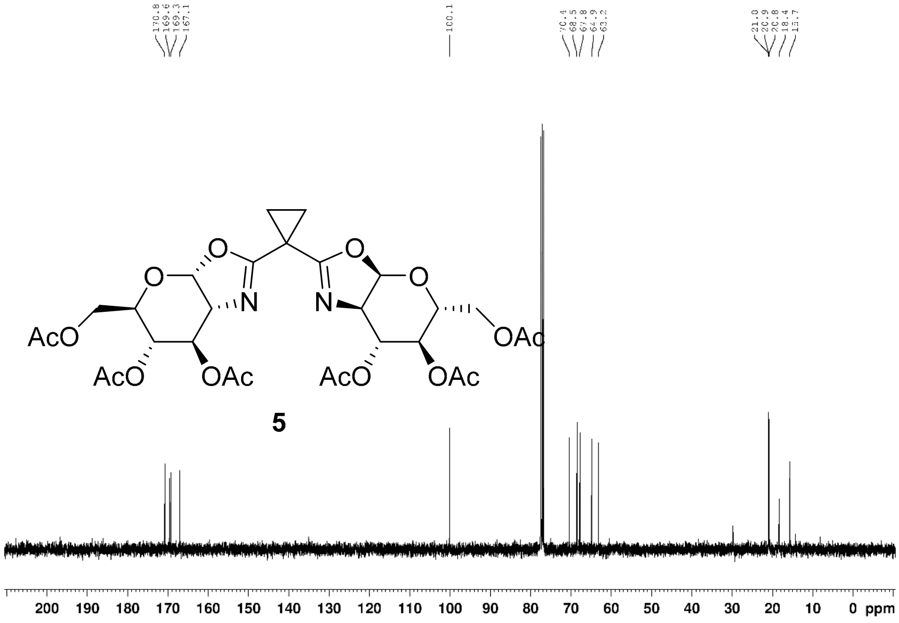

Figure S6. ^{13}C-NMR of compound **5**.

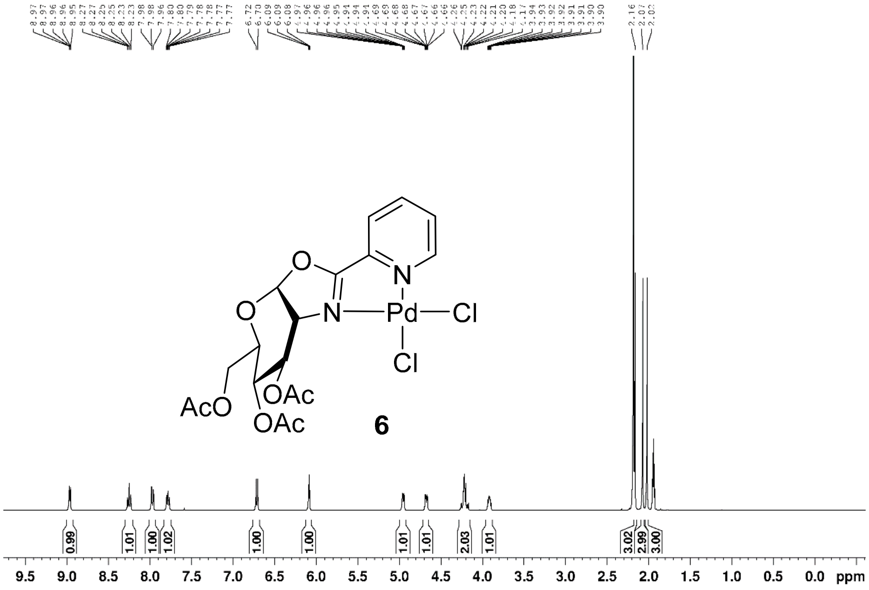

Figure S7. ^{1}H-NMR of compound **6**.

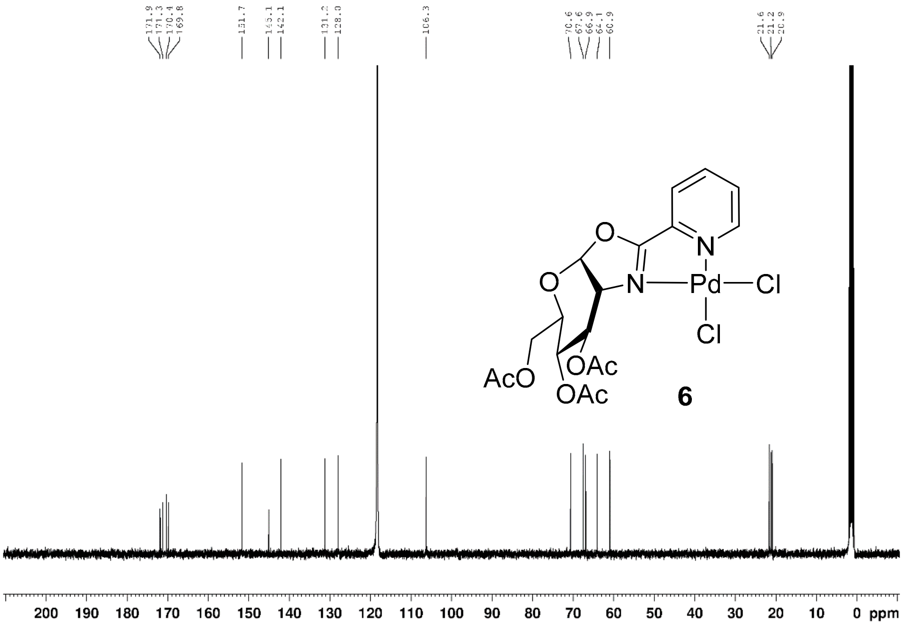

Figure S8. ^{13}C-NMR of compound **6**.

Crystal Structure Data

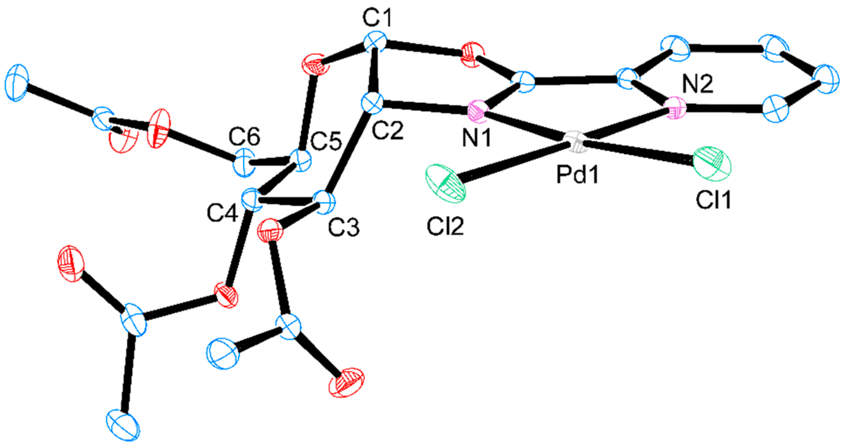

Figure S9. ORTEP plot of the molecular structure of Pd-complex **6**. Hydrogen atoms have been omitted for clarity; ellipsoids are given at the 50% probability level. Blue = carbon; red = oxygen; pink = nitrogen; grey = palladium; green = chlorine.

Table 1. Crystal data and structure refinement for mo_jk268_0m.

**Identification Code**	**mo_jk268_0m**
Empirical formula	$C_{18}H_{20}Cl_2N_2O_8Pd$
Formula weight	569.66
Temperature	100(2) K
Wavelength	0.71073 Å
Crystal system	Orthorhombic
Space group	$P\ 2_1\ 2_1\ 2_1$
Unit cell dimensions	a = 8.6185 (6) Å, α= 90°<br>b = 14.6441 (10) Å, β= 90°.<br>c = 16.5033 (11) Å, γ = 90°.
Volume	2082.9(2) Å3
Z	4
Density (calculated)	1.817 Mg/m^3
Absorption coefficient	1.197 mm^{-1}
F(000)	1144
Crystal size	0.262 × 0.119 × 0.073 mm^3
Theta range for data collection	2.666 to 30.526°.
Index ranges	$-12 \leq h \leq 12$, $-20 \leq k \leq 20$, $-23 \leq l \leq 23$
Reflections collected	44885
Independent reflections	6370 [R(int) = 0.0287]
Completeness to theta = 25.242°	99.9%
Absorption correction	Semi-empirical from equivalents
Max. and min. transmission	1.00000 and 0.8893
Refinement method	Full-matrix least-squares on F^2
Data / restraints / parameters	6370/0/283
Goodness-of-fit on F2	1.059
Final R indices [I>2sigma(I)]	R1 = 0.0144, wR2 = 0.0360
R indices (all data)	R1 = 0.0148, wR2 = 0.0362
Absolute structure parameter	−0.008(5)
Extinction coefficient	n/a
Largest diff. peak and hole	0.350 and −0.326 e·Å^{-3}

Table 2. Atomic coordinates (×10^4) and equivalent isotropic displacement parameters (Å^2 × 10^3) for mo_jk268_0m. U(eq) is defined as one third of the trace of the orthogonalized U^{ij} tensor.

Atom	x	y	z	U(eq)
C(1)	10535(2)	3556(1)	9028(1)	13(1)
C(2)	10269(2)	3110(1)	9872(1)	12(1)
C(3)	9167(2)	3640(1)	10432(1)	12(1)
C(4)	9137(2)	4654(1)	10246(1)	13(1)
C(5)	8826(2)	4786(1)	9343(1)	13(1)
C(6)	9309(2)	2227(1)	8883(1)	11(1)
C(31)	8607(2)	3255(1)	11810(1)	14(1)
C(32)	9280(2)	3179(1)	12643(1)	19(1)
C(41)	8302(2)	5624(1)	11332(1)	17(1)
C(42)	6965(2)	5758(2)	11895(1)	23(1)
C(51)	8634(2)	5773(1)	9089(1)	16(1)
C(52)	10055(2)	7150(1)	9264(1)	16(1)
C(53)	11419(2)	7549(1)	9703(1)	24(1)
C(61)	8719(2)	1410(1)	8481(1)	11(1)
C(62)	8353(2)	1362(1)	7666(1)	16(1)
C(63)	7806(2)	535(2)	7361(1)	19(1)
C(64)	7649(2)	−199(1)	7878(1)	20(1)
C(65)	8076(2)	−113(1)	8692(1)	16(1)
N(1)	9645(2)	2204(1)	9645(1)	11(1)
N(2)	8610(2)	680(1)	8987(1)	12(1)
O(1)	10201(2)	4460(1)	8932(1)	16(1)
O(2)	9542(2)	2996(1)	8482(1)	13(1)
O(6)	9553(2)	5972(1)	11386(1)	26(1)
O(8)	7274(2)	3106(1)	11637(1)	21(1)
O(31)	9680(1)	3530(1)	11259(1)	13(1)
O(41)	7900(2)	5031(1)	10722(1)	15(1)
O(51)	9900(2)	6264(1)	9456(1)	22(1)
O(52)	9216(2)	7538(1)	8798(1)	25(1)
Cl(1)	9247(1)	−552(1)	10515(1)	20(1)
Cl(2)	10319(1)	1345(1)	11369(1)	21(1)
Pd(1)	9427(1)	937(1)	10127(1)	11(1)

Table 3. Bond lengths [Å] and angles [°] for mo_jk268_0m.

C(1)-O(1)	1.364(2)
C(1)-O(2)	1.489(2)
C(1)-C(2)	1.556(2)
C(1)-H(1)	1.0000
C(2)-N(1)	1.479(2)
C(2)-C(3)	1.536(2)
C(2)-H(2)	1.0000
C(3)-O(31)	1.444(2)
C(3)-C(4)	1.518(2)
C(3)-H(3)	1.0000
C(4)-O(41)	1.435(2)
C(4)-C(5)	1.526(3)
C(4)-H(4)	1.0000
C(5)-O(1)	1.447(2)
C(5)-C(51)	1.513(3)
C(5)-H(5)	1.0000
C(6)-N(1)	1.290(2)
C(6)-O(2)	1.321(2)

Table 3. *Cont.*

C(6)-C(61)	1.461(2)
C(31)-O(8)	1.204(2)
C(31)-O(31)	1.358(2)
C(31)-C(32)	1.496(3)
C(32)-H(32A)	0.9800
C(32)-H(32B)	0.9800
C(32)-H(32C)	0.9800
C(41)-O(6)	1.196(2)
C(41)-O(41)	1.373(2)
C(41)-C(42)	1.493(3)
C(42)-H(42A)	0.9800
C(42)-H(42B)	0.9800
C(42)-H(42C)	0.9800
C(51)-O(51)	1.440(2)
C(51)-H(51A)	0.990000
C(51)-H(51B)	0.990000
C(52)-O(52)	1.199(2)
C(52)-O(51)	1.343(2)
C(52)-C(53)	1.499(3)
C(53)-H(53A)	0.9800
C(53)-H(53B)	0.9800
C(53)-H(53C)	0.9800
C(61)-N(2)	1.359(2)
C(61)-C(62)	1.382(2)
C(62)-C(63)	1.393(3)
C(62)-H(62)	0.9500
C(63)-C(64)	1.379(3)
C(63)-H(63)	0.9500
C(64)-C(65)	1.399(3)
C(64)-H(64)	0.9500
C(65)-N(2)	1.341(2)
C(65)-H(65)	0.9500
N(1)-Pd(1)	2.0284(14)
N(2)-Pd(1)	2.0439(15)
Cl(1)-Pd(1)	2.2779(5)
Cl(2)-Pd(1)	2.2692(5)
O(1)-C(1)-O(2)	110.11(14)
O(1)-C(1)-C(2)	118.68(14)
O(2)-C(1)-C(2)	103.08(12)
O(1)-C(1)-H(1)	108.2
O(2)-C(1)-H(1)	108.2
C(2)-C(1)-H(1)	108.2
N(1)-C(2)-C(3)	112.38(13)
N(1)-C(2)-C(1)	101.68(13)
C(3)-C(2)-C(1)	114.65(14)
N(1)-C(2)-H(2)	109.3
C(3)-C(2)-H(2)	109.3
C(1)-C(2)-H(2)	109.3
O(31)-C(3)-C(4)	107.75(13)
O(31)-C(3)-C(2)	108.83(13)
C(4)-C(3)-C(2)	112.57(14)
O(31)-C(3)-H(3)	109.2
C(4)-C(3)-H(3)	109.2
C(2)-C(3)-H(3)	109.2

Table 3. *Cont.*

O(41)-C(4)-C(3)	106.10(14)
O(41)-C(4)-C(5)	110.84(13)
C(3)-C(4)-C(5)	108.92(14)
O(41)-C(4)-H(4)	110.3
C(3)-C(4)-H(4)	110.3
C(5)-C(4)-H(4)	110.3
O(1)-C(5)-C(51)	105.98(14)
O(1)-C(5)-C(4)	105.74(14)
C(51)-C(5)-C(4)	114.21(15)
O(1)-C(5)-H(5)	110.2
C(51)-C(5)-H(5)	110.2
C(4)-C(5)-H(5)	110.2
N(1)-C(6)-O(2)	118.48(15)
N(1)-C(6)-C(61)	119.97(15)
O(2)-C(6)-C(61)	121.53(14)
O(8)-C(31)-O(31)	123.02(17)
O(8)-C(31)-C(32)	125.06(17)
O(31)-C(31)-C(32)	111.91(15)
C(31)-C(32)-H(32A)	109.5
C(31)-C(32)-H(32B)	109.5
H(32A)-C(32)-H(32B)	109.5
C(31)-C(32)-H(32C)	109.5
H(32A)-C(32)-H(32C)	109.5
H(32B)-C(32)-H(32C)	109.5
O(6)-C(41)-O(41)	123.49(18)
O(6)-C(41)-C(42)	126.32(17)
O(41)-C(41)-C(42)	110.19(16)
C(41)-C(42)-H(42A)	109.5
C(41)-C(42)-H(42B)	109.5
H(42A)-C(42)-H(42B)	109.5
C(41)-C(42)-H(42C)	109.5
H(42A)-C(42)-H(42C)	109.5
H(42B)-C(42)-H(42C)	109.5
O(51)-C(51)-C(5)	106.14(14)
O(51)-C(51)-H(51A)	110.5
C(5)-C(51)-H(51A)	110.5
O(51)-C(51)-H(51B)	110.5
C(5)-C(51)-H(51B)	110.5
H(51A)-C(51)-H(51B)	108.7
O(52)-C(52)-O(51)	123.31(18)
O(52)-C(52)-C(53)	126.77(18)
O(51)-C(52)-C(53)	109.91(17)
C(52)-C(53)-H(53A)	109.5
C(52)-C(53)-H(53B)	109.5
H(53A)-C(53)-H(53B)	109.5
C(52)-C(53)-H(53C)	109.5
H(53A)-C(53)-H(53C)	109.5
H(53B)-C(53)-H(53C)	109.5
N(2)-C(61)-C(62)	122.84(16)
N(2)-C(61)-C(6)	112.86(14)
C(62)-C(61)-C(6)	124.26(16)
C(61)-C(62)-C(63)	118.20(18)
C(61)-C(62)-H(62)	120.9
C(63)-C(62)-H(62)	120.9

Table 3. *Cont.*

C(64)-C(63)-C(62)	119.14(17)
C(64)-C(63)-H(63)	120.4
C(62)-C(63)-H(63)	120.4
C(63)-C(64)-C(65)	119.88(18)
C(63)-C(64)-H(64)	120.1
C(65)-C(64)-H(64)	120.1
N(2)-C(65)-C(64)	121.09(18)
N(2)-C(65)-H(65)	119.5
C(64)-C(65)-H(65)	119.5
C(6)-N(1)-C(2)	107.79(14)
C(6)-N(1)-Pd(1)	112.68(11)
C(2)-N(1)-Pd(1)	138.95(11)
C(65)-N(2)-C(61)	118.80(15)
C(65)-N(2)-Pd(1)	127.65(13)
C(61)-N(2)-Pd(1)	113.46(11)
C(1)-O(1)-C(5)	116.08(14)
C(6)-O(2)-C(1)	104.64(12)
C(31)-O(31)-C(3)	117.20(13)
C(41)-O(41)-C(4)	117.19(14)
C(52)-O(51)-C(51)	117.34(15)
N(1)-Pd(1)-N(2)	80.70(6)
N(1)-Pd(1)-Cl(2)	94.69(4)
N(2)-Pd(1)-Cl(2)	175.28(4)
N(1)-Pd(1)-Cl(1)	173.04(4)
N(2)-Pd(1)-Cl(1)	93.43(4)
Cl(2)-Pd(1)-Cl(1)	91.230(18)

Symmetry transformations used to generate equivalent atoms:

Table 4. Anisotropic displacement parameters ($Å^2 \times 10^3$) for mo_jk268_0m. The anisotropic displacement factor exponent takes the form: $-2\pi^2[h^2a^{*2}U^{11} +, ..., + 2hka^*b^*U^{12}]$.

**Atom**	**U11**	**U22**	**U33**	**U23**	**U13**	**U12**
C(1)	11(1)	13(1)	15(1)	−1(1)	2(1)	−2(1)
C(2)	10(1)	12(1)	13(1)	−3(1)	1(1)	−1(1)
C(3)	11(1)	15(1)	11(1)	−2(1)	−1(1)	−1(1)
C(4)	10(1)	13(1)	16(1)	−5(1)	−1(1)	1(1)
C(5)	12(1)	12(1)	16(1)	−3(1)	1(1)	−1(1)
C(6)	10(1)	13(1)	11(1)	0(1)	1(1)	1(1)
C(31)	14(1)	14(1)	14(1)	−3(1)	2(1)	−1(1)
C(32)	18(1)	27(1)	13(1)	−4(1)	1(1)	−3(1)
C(41)	20(1)	16(1)	16(1)	−5(1)	−5(1)	5(1)
C(42)	23(1)	26(1)	19(1)	−7(1)	1(1)	6(1)
C(51)	16(1)	14(1)	19(1)	−2(1)	−4(1)	1(1)
C(52)	18(1)	13(1)	17(1)	−3(1)	4(1)	−1(1)
C(53)	24(1)	16(1)	32(1)	−5(1)	−7(1)	−3(1)
C(61)	9(1)	14(1)	11(1)	−2(1)	1(1)	1(1)
C(62)	15(1)	21(1)	12(1)	−2(1)	−1(1)	3(1)
C(63)	15(1)	27(1)	14(1)	−9(1)	−3(1)	3(1)
C(64)	15(1)	22(1)	22(1)	−10(1)	0(1)	−2(1)
C(65)	15(1)	15(1)	19(1)	−4(1)	2(1)	−1(1)
N(1)	10(1)	12(1)	10(1)	−1(1)	0(1)	0(1)
N(2)	10(1)	14(1)	12(1)	−2(1)	1(1)	−1(1)

Table 4. *Cont.*

Atom	U11	U22	U33	U23	U13	U12
O(1)	15(1)	12(1)	20(1)	0(1)	6(1)	−1(1)
O(2)	15(1)	12(1)	12(1)	1(1)	0(1)	−1(1)
O(6)	22(1)	29(1)	28(1)	−14(1)	−4(1)	−1(1)
O(8)	13(1)	30(1)	21(1)	1(1)	1(1)	−5(1)
O(31)	11(1)	19(1)	10(1)	−3(1)	0(1)	−2(1)
O(41)	12(1)	17(1)	16(1)	−7(1)	−1(1)	3(1)
O(51)	24(1)	12(1)	29(1)	1(1)	−12(1)	−4(1)
O(52)	28(1)	19(1)	27(1)	5(1)	−7(1)	−3(1)
Cl(1)	28(1)	15(1)	18(1)	4(1)	7(1)	4(1)
Cl(2)	26(1)	26(1)	11(1)	−4(1)	−4(1)	11(1)
Pd(1)	11(1)	12(1)	9(1)	0(1)	1(1)	2(1)

Table 5. Hydrogen coordinates (×10^4) and isotropic displacement parameters (Å^2 × 10^3) for mo_jk268_0m.

	x	y	z	U(eq)
H(1)	11643	3457	8871	16
H(2)	11292	3028	10150	14
H(3)	8094	3386	10376	15
H(4)	10148	4942	10399	16
H(5)	7900	4421	9174	16
H(32A)	9562	3788	12840	29
H(32B)	10208	2793	12626	29
H(32C)	8512	2907	13008	29
H(42A)	7302	6119	12363	34
H(42B)	6586	5162	12080	34
H(42C)	6130	6081	11612	34
H(51A)	8675	5828	8492	20
H(51B)	7627	6016	9281	20
H(53A)	12380	7352	9438	36
H(53B)	11416	7337	10266	36
H(53C)	11352	8217	9691	36
H(62)	8472	1878	7324	19
H(63)	7544	478	6804	23
H(64)	7253	−762	7681	23
H(65)	7985	−625	9043	20
